# Isotonic Sea Water in Therapeutics*Read before Wayne County Medical Society, Detroit, Mich., Dec. 16, 1912.

**Published:** 1913-02

**Authors:** Rollin H. Stevens

**Affiliations:** Dermatologist and Pœntgenologist to Grace Hospital and German Polyclinic Detroit; 623 Stevens Building


### BUFFALO MEDICAL JOURNAL

VOLUME 68 FEBRUARY, 1913 No. 7

### ORIGINAL ARTICLES

### Isotonic Sea Water in Therapeutics.*

By DR. ROLLIN H. STEVENS
Dermatologist and Roentgenologist to Grace Hospital and German Polyclinic
Detroit

IN these days of modern medicine with its very high standards of education, its therapeutic nihilism and its therapeutic numerosity, with its consequent quackery and multiplicity of medical sects, with its scientific skepticism and its psychic research, one who, at least thinks he thinks, becomes somewhat Oslerian and hesitates to give full credit to any therapeutic method, which may theoretically and practically appear to be of great value.

And so when there is introduced a new therapeutic method of such wide applicability as isotonic sea water, the principles of which are new to some, it is done with considerable hesitancy, with a realizing sense that the case is not proved, and that the jury will disagree. However, it may be decided the facts herein presented are at least worthy of most serious consideration.

For whatever of benefit there may be derived from the use of isotonic sea water in therapeutics, the world is indebted to Dr. Rene Quinton, assistant in the Laboratory of Pathological Physiology in the College of France.

Quinton, who is a biologist, and not a practitioner of medicine, in the year 1897 presented to the Societe de Biologie three papers concerning the action of sea water. These were followed in the next year by two more, one entitled "The Amoeboid Movements of White Blood Corpuscles in a Marine Dilution," and the other, "Constancy of the Marine Medium as a Vital Medium Throughout the Animal Series." In 1904 he published a book of some five hundred pages entitled "Sea Water the Organic Medium." A second edition of this book appeared this year.

Since then Quinton, Simon, Fumeux, Arnulphy and other writers have contributed to the literature of the subject in the French journals.

As early as 1900 two papers were presented to the International Congress for Tuberculosis in Paris on the treatment of tuberculosis by sea water.

* Read before Wayne County Medical Society, Detroit, Mich., Dec. 16, 1912.

The evolution of the theories and demononstration of the laws upon which sea water therapeutics are founded, represent on the part of Dr. Quinton, I understand, some twenty years of study and research in the Marine Biological Laboratories at Arachnon and in the Laboratories of the College of France.

Before describing the treatment and its results, we shall review briefly Quinton's theories, demonstrations, and laws, in order to better appreciate the foundation on which the therapeutics rest.

1. *Aquatic Origin.*—The aquatic origin of every animal organism is accepted because it derives its origin from a primary cell, which being mostly liquid enclosed by an osmotic membrane must necessarily be aquatic; because, after the cellular stage, the first stages thru which typical embryos of all animal groups pass, are stages clearly aquatic, and because of the convincing results of the study of the four different modes of respiration in animals, namely, the cellular, tegumentary, branchial and tracheal, which we shall not discuss at this time.

2. *The Marine Origin of the First Animal Cells.*—All the animal organisms are derived from marine animal organisms, and every organism is derived from a cell, so, it naturally follows, that the original cell was of Marine origin.

Again today there is a considerable number of marine organisms which are derived from marine cells by processes of development which are the least differentiated primitive processes, processes therefore which would be proper for the ancestral organisms of all animal stocks.

Therefore, the ancestral cells from which have been derived all animal organisms have been marine cells.

3. *The Maintenance of the Original Marine Medium as the "Vital Medium" of the Cells.*—What is the "Vital Medium?" It is the totality of the plasmas of the cranial, peritoneal, pleuritic, pericardic and joint cavities; of the interstitial lymph; of the lymph channels; of the haemolymph or blood; of the plasmas of imbition present as a result of osmosis in epithulium, endothelium, etc., of the different fundamental structural substances in cells; of the basal membranes and intercellular cement; of connective tissue and cartilage, etc.

Quinton discards the old histological divisions of the organism which are founded on the notion of tissue—namely, the epithelial, nervous, muscular connective cartilaginous osseous blood, lymphatic, etc. This division does not separate the Vital Medium from that which is not Vital medium.

He substitutes for the old classification four grand divisions, viz:

1. Vital Medium which has been described.
2. Living Matter.
3. Dead Matter.
4. Secreting Matter.

Living Matter is composed of the whole of the cells endowed with the life of the organism according to the organ to which they belong.

Dead Matter is so called because it partakes of none of the characteristics of the living matter. It is truly inert matter; DEAD, tho of living origin, and is the ensemble of all cellular productions destined to play in the organism a role purely physical or mechanical.

It may be for union or for isolation of cells, such as the fundamental substance of epithelium, connective tissue, cartilage, etc. It may be for architectural support, such as the fundamental substance of dense connective, cartilaginous and bony tissues, connective tissue of fasciuli and of elastic fibres. It may be for attack or protection, as the fundamental substance of dental tissue, epithelial covering of the whole ectoderm, etc.

Secreting Matter is composed of the various secreting cells of the organism. The vital medium through the processes of osmosis and diffusion, provides the living secreting matter with the elements necessary for their proper functioning growth and development.

By many personal and numerous reported authorities, chemical analysis, by comparative biological examination, and by physiological experiment, Quinton establishes the close similarity of the Vital medium thru out the animal series with the original sea water in which animal life first appeared, and the sea water of today, due allowance being made for evaporation and condensation in the process of cooling of the earth's surface.

The chemical analyses are not yet complete, the detection of some elements probably present in infinitesimal amounts being somewhat difficult with our present knowledge.

He has shown, however, that these infinitesimal amounts are absolutely essential to the functioning and life of the whole organism, that the importance of any element cannot be gauged by any means, by the mere proportion in which it appears in the organism.

The few milligrams of iodine present in the whole body of a human being and confined to the thyroid gland are absolutely essential, not only to health and well being, but also to life itself. Those few milligrams of iodine are thus just as important to the animal mechanism as the Sodium Chloride which predominates over the mineral composition of the plasma. The same is true of the infinitesimal quantity of arsenic and other elements present only in infinitesimal amount.

The kinetic energy set free from the potential is often out of all proportion to the transforming agent. The friction of a match or a spark of electricity is sufficient to transform enough energy in powder to destroy a powder magazine or move a mountain.

By variations of pressure of less than a thousandth of a millimeter, different degrees of penetration in the X-Rays are produced. And so it would seem to be quite impossible to make an artificial sea water or Vital Medium, because the leaving out of an infinitesimal which appears to be essential to the natural properties of the whole would be as serious a blunder as leaving out the sodium and chlorine.

In vertebrates as well as in invertebrates direct chemical analysis confirms the identity of the "Vital Medium" and the Marine medium. Chemical analysis of modern sea water shows it to be composed as follows:

| | |
|---|---|
| Water .......... | 1000 |
| Dissolved Salts... | 35 |

These salts are divided into four great groups of decreasing importance as follows:

| | | | Hundredths of The Solution. |
|---|---|---|---|
| 1. | Chlorine<br>Sodium | forming for them alone............. | 84. |
| 2. | Sulphur<br>Magnesium<br>Potassium<br>Calcium | forming altogether................ | 14. |
| 3. | Bromine, Carbon, Silicon, Ferrun, Nitrogen, Ammonia, Fluoricum, Phosphorus, Lithium, Iodine, Borium.. | forming altogether almost the totality of the remaining salts... | 1.9997 |
| 4. | Arsenic, Copper, Silver, Gold, Zinc, Manganese, Strantium, Barium, Coesium, Rhubidium, Aluminum, Lead, Cobalt......... | All these bodies only constituting together an infinitesimal part of the dissolved mass | 0.0003 |
| | | | 100.00 |

Quinton, however, to maintain his thesis, "the constancy of the original marine medium of the cells" rightly argues that the chemical composition of the modern sea does not matter so much, but we should know the composition of the ancient sea in which animal life appeared, which was not until after the earth cooled to 44 or 45 C. or the precambrian period. Portions of the ancient seas being isolated came to an end by being separated completely from the oceanic mass. Evaporation of their waters kept them solid banks of saline deposits which we use and study today in the many mineral springs in different parts of the world. We have no deposits of the precambrian period, but those at the end of the primary (Permian) period and beginning of the secondary (Triassic) period are numerous. These are relatively near the precambrian seas in point of time, and the analysis of these deposits shows a striking analogy between the composition of the ancient and modern seas.

There is not time to go into any details concerning the hundreds of analyses of the waters of the ancient and modern seas, nor of the "Vital Medium." Suffice it to say that they approximate very closely the analyses just given for the modern sea water, all the elements mentioned, except gold and cobalt, being found, directly or indirectly, in the plasma or "vital medium" of the animal economy.

It may be asked, "Do not these elements gain entrance to the organism through the food and drink and their amount vary with the alimentation? This does not appear to be likely for superior vertebrates are nourished by vegetables extremely poor in chlorine and in sodium.

To be sure there exist some inferior organisms which have not conserved their original marine medium, such, for instance, as the Anodonta cygnea (fresh water mussel), fresh water hydra, etc. They are open osmotically to the ambiant medium, but these organisms seem to be manifestly in a state of degeneration.

The more and more complicated and independent organisms inhabiting the border of the sea, then living afterwards in the fresh waters and on the land, have always tended to preserve their component cells in a natural or modified marine medium.

For instance, in the primary organisms of the animal series the porifera, hydrozoa and scyphozoa, the internal vital medium is sea water itself. It penetrates the entire organism and bathes all the cells, being carried there by the innumerable canaliculi, analagous to the capillaries.

In the higher marine invertebrates the external covering of the animal is permeable to the water and salt, so that the internal vital medium of the animal is from the mineral point of view a marine medium.

Out of a total of 15 groups, 23 branches, 61 classes, 228 orders composing the animal kingdom, the marine invertebrates constitute of themselves alone 12 groups, 18 branches, 46 classes, 43 orders.

In the fresh water invertebrates the animal is neither permeable to water nor to salts and preserves in an external medium almost totally unsalted its vital medium, which is in a very high degree saline, specific constant, and always analagous to the sea medium.

Finally, in the highest organisms of the geological series, the vertebrates, the farthest removed from the marine stock, experiment and chemistry established the identity of the vital medium of the cells and the sea medium, as witness these experiments of Quinton's:

Three dogs were injected with isotonic sea water, the first with 66 per cent. of its weight, another with 81 per cent. and the third with 104 per cent., without anyone of them presenting the least trouble.

Again, he almost completely exsanguinated two dogs, and at the moment when they were about to succumb, he injected a quantity of isotonic sea water equal to that of the blood lost. The following day the dogs ran about, and in five days they had recovered their lost haemoglobin.

We took a unit of blood from all classes of vertebrates (fish, batrachians, reptiles, mammifera, birds) mixed it with varying quantities of sea water, namely, 25, 50 and 100 times greater, and established that the white corpuscles live with all the appearances of a normal existence, when it is impossible to preserve them in any other foreign medium, save normal salt solution, and in that they live only a few hours at most.

Quinton, as a result of his observations and experimentations, announced the following laws which form the basis for sea water therapeutics:—

### Law of Original Marine Constancy.

"Animal life, which appeared in the cellular state in the sea, has tended to maintain for its high cellular functioning throughout the zoological series, the cells composing each organism in a marine medium. It has not maintained this medium in all organisms, but those in which the maintenance was not effected have undergone a vital decadence."

### Law of Thermic Constancy.

"In the face of the cooling of the globe animal life which appeared in the cellular state with a determined temperature, has tended to maintain for its high cellular functioning, in the organisms indefinitely created to this end, that temperature of origin."

### Law of Osmotic or Saline Constancy.

"In the face of progressive concentration of the oceans, animal life which appeared in the cellular state in seas of determined saline concentration, has tended to maintain thruout the zoological series for its high cellular function that concentration of origin."

### General Law of Original Constancy.

"In the face of variations of every order which its different habitats in the course of ages can undergo, animal life, evident in the cellular state in determined physical and chemical conditions, tends to maintain for its high cellular functioning throughout the geological series, these conditions of origin."

Quinton comments on the general law of original constancy thus:—

"This law shows what modern science has endeavored to ignore —that life is a phenomenon subject to sufficiently narrowly determined conditions, for, since its origin, in spite of the time that has passed, in spite of combat, in spite of the causes of variation, life does not appear to have done better than to maintain invariable for its high activity the conditions of origin."

In 1906 there was established by one of the children's societies in Paris a dispensary for the treatment of disease by subcutaneous injections of isotonic sea water. A short time later there was established another similar dispensary in another part of the city. And now there are several such dispensaries in Paris and other French cities.

In 1909 it was my privilege to visit these dispensaries and investigate the claims made for the treatment. I was so impressed with the apparent success of the method that I brought home a supply of the prepared isotonic sea water in sealed ampules and began the treatment in my private practice, and am still continuing it.

### Source of Supply.

Through the courtesy of Mr. George M. Gray, Curator of the Marine Biological Laboratory at Wood's Hole, Mass., fresh sea water captured many miles from the coast, and at considerable depth, in sterile carboys has been received about every month.

This, after filtering through filter paper, is diluted in the proportion of two parts of sea water to five parts of freshly distilled water, and this is then twice filtered through a germ proof Chamber and filter into sterile glass receptacles ready for use by subcutaneous injections. Of course sea water for this purpose cannot be sterilized in any other manner without changing materially its composition. Indeed, Quinton showed that sea water heated in the autoclave one-half hour at 120 C. was so toxic that when 700 c.c. were injected into a dog of 10 kgr. weight, it died in a few days. He was never able to obtain amoeboid movements in white blood corpuscles placed in sea water mixture thus sterilized. At 105° these effects are attenuated rapidly. Sea water kept in this temperature for ten minutes only gave excellent results. Lead glass containers destroy its therapeutic value.

The container is fitted with three feet of pure gum rubber tubing with heavy wall and a lumen of about one-eighth inch and a platinum needle of about No. 20 gauge, both of which latter have been thoroughly boiled. After sterilizing the skin of the buttock, just behind the great trochanter, where the injections should be made, and after making sure that all water in which the tube was boiled has run out, and that all air is forced out, the needle with a quick thrust is driven straight into the tissues for about an inch. This is usually quite painless if done with dispatch. The sea water then runs in slowly while the patient remains in the sitting or recumbent position. It is better when several injections are given the same patient, to inject in the same place each time as the local pain following the first injection is much attenuated in the later ones.

### Dose.

Quinton advises that an amount equal to about 1/100 or 1/150 of the body weight of the patient be given as the minimum dose. For instance, a man weighing 150 pounds should receive about 700 c. c.

In the dispensaries in Paris, however, I noted they usually gave no more than 50 to 200 c. c. to an adult of such weight and larger doses proportionately, 30 to 75 c. c. to children, who seem to bear relatively larger amounts with less reaction.

In my practice doses of varying size from 30 c. c. to 800 c. c. for an adult have been tried and the larger doses appear to give better results, namely, 200 to 700 c. c. for the adult, repeated every five to ten days. The dose and interval of repetition will depend somewhat on the condition being treatd. This will be discussed later.

Quinton advises using fresh sea water. It loses its properties after varying lengths of time—a few weeks—and becomes relatively toxic. In psoriasis, however, I have recently been informed by Dr. Arnulphy of Paris that the older the sea water is the better. In the last three months I have tried this out on a few cases with better success.

### Immediate Effects of the Injection.

Depending upon the size of the dose and the susceptibility of the patient following the injection there will be a reaction more or less severe. As the water is running in there is usually no pain. Immediately after the hip feels full and heavy. In a variable period after, usually about four hours, but it may be before the injection is completed, or within one or two hours after, there is frequently a chill more or less pronounced lasting a few minutes to an hour or more. This is accompanied by a rise of temperature of one and one-half to two, or even four degrees. There is likely to be a little nausea and some aching of the limbs, head and back. This may only last a couple of hours, but usually about twelve hours, and may be prolonged to forty-eight hours. There is thirst and often increased urination.

In one case a thin, overworked woman of about 90 pounds who had had gonorrhoial infection and several abdominal operations, the reaction on several occasions were severe after the use of 50 c. c. to 75 c. c. and was accompanied by sneezing and a severe coryza lasting 24 to 48 hours. With 30 c. c. the reaction was practically nil.

In my own case my weight being 170 pounds, and being in fairly good health, I had a reaction with temperature of 102.5 and slight nausea for 48 hours after an injection of 50 c. c. This is exceptional with so small a dose.

In many cases there is no perceptible reaction to such small doses as 50 c. c.

### Indications for Treatment by Isotonic Sea Water.

On this division of the subject Quinton writes the following, which we translate freely:—"We have today in the organism a veritable marine aquarium, a conception which was wanting yesterday. An organism is composed of living cells, all situated in contact with a liquid, which we have called their vital medium and which is a marine liquid.

"Let us imagine a culture tube and in this culture tube sea water; in this sea water and growing there organic cells; there is the scheme of an organism. If we remember the importance for a culture of the liquid in which it grows, we behold the rank which sea water therapeutics can take in all cases where the culture liquid of the organic cells (vital medium) is vitiated for any cause whatsoever, chemical or microbe poisoning, insufficiency of the emunctories, defects of certain elementary products, etc.

And so he tried it in some acute cases of particular gravity, infectious gastro entritis of undetermined nature, poisoning by oxalic acid, erysipelas, etc., and the success was immediate and complete.

In precocious and malignant syphilis and cutaneous tuberculosis, cicatrization of the ulcers promptly occurred under administration of sea water.

### Results Observed at the Marine Dispensaries in Paris in 1909.

In my visits to the Dispensaires Marins of Paris I was cordially shown the patients, and a large number of photographs and chemical histories. The cases in which they seemed to have greatest success where those of gastro-enteritis and marasmus, those conditions formerly classed as scrofula, tuberculosis in all forms, rachitis, anaemia, dysmennorrhoea, constipation, varicose ulcers, neurasthenia and many skin diseases, especially eczema, psoriasis and lupus.

Some of the results in gastro-enteritis and marasmus in children seemed almost miraculous, and the transformation in their appearance in a month's time seemed hardly credible. Improvement began within a day or two, and though the children were at once put on their regular diet, the functioning of the stomach and bowels seemed to improve very rapidly, and in a month or six weeks, old-looking, scrawny, dried up marasmic babies at the point of death and living under most unhygienic conditions, often became plump and well.

The pain of dysmennorrhoea was relieved very promptly. Constipation was improved and often cured by this method. Old tuberculosis sinuses in joints and bones, as well as varicose ulcers, were healed after a few weeks or months without the intervention of operations and without local treatment. Many cases of eczema, particularly in infants, promptly improved and were finally cured, as were a few cases of psoriasis, while the results in lupus seemed to rival those at the Finsen Institute in Copenhagen, though the number treated was very much smaller.

In our personal experience in the use of sea water during the past three years we have met with considerable success and some disappointment. No case has been injured by the treatment and many have been improved. While my therapeutic practice has been limited to dermatology, I have directed the treatment of some hospital cases suffering from various diseases other than those of the skin where it seemed to me the treatment was indicated and where I wanted to prove up what I saw in Paris.

We have used sea water as an adjuvant or alone with more or less success in the following diseases: Eczema, psoriasis, pityriasis rosea, lupus vulgaris, mycosis fungoides, epithelioma of the skin and cancer of other organs, tuberculosis of glands and joints, gangrenous dermatitis, dysmennorrhoea, infectious eczematoid dermatitis, acne vulgaris, seborrhoeic dermatitis, rosacea, tertiary syphilis, varicose ulcer, keratodermia, erythema multiforme, T. B. of skin and bones, purpura simplex, general pruritus, urticaria facitia, etc.

We have treated one case of lupus vulgaris of the face by a combination of sea water and Finsen light. The case was a young woman, 23 years old. She had had the lupus for several years, had been operated upon one year previously for tubercular peritonitis and had quite large tubercular cervical glands. Two treatments with Finsen light were given ten days apart. During this period she also had subcutaneous injections of sea water, about 75 to 100 c. c., twice a week. At the end of ten days the lupus was almost healed and in three weeks no characteristic apple jelly spots could be detected in it. The sea water was continued for eight months and X rays given the tubercular glands. The latter improved rapidly, but two remained about the size of a pea and a bean, respectively, for over a year, the X-ray being given them occasionally. The patient gained much in weight, and after two years there has been no relapse of the lupus.

Results of treatment of lupus with the Finsen light after a long experience in Copenhagen and in my private practice, while gratifying, are not usually so prompt and perfect as in this case, so that we feel justified in ascribing a large part of the victory to the sea water.

A young woman, a music teacher, 20 years old, had suffered with pityriasis of the face for a couple of years. She was badly nourished and overworked; she was very nervous and suffered severely from dysmennhorrhoea, being confined to bed the first day or two each month. She had had a currettage without gaining any relief. She began taking sea water two weeks before her menses were due. She was given 50 to 75 c. c. every third day. The next menstruation was so nearly painless, she kept at her work during the whole period. Subsequent menstruations for about a year and a half were almost wholly free from pain. She has not reported for over a year. The pityriasis was much improved, though she had some local treatment for that, and her general condition was very satisfactory, after about three months treatment. The treatments were continued off and on for 13 months, 67 treatments in all being given.

Four cases of psoriasis were treated by sea water. One, an acute general exacerbation, promptly subsided in six weeks' treatment with doses of 50 to 100 c. c. of fresh sea water twice a week. She was not cured, however, when last seen, and there were still some slight scaly patches on the elbows. Two others showed marked improvement under injections of a pint to a pint and one-half of sea water, three or four months old, at irregular intervals of 10 days to two or three weeks. But these cases have been under treatment off and on for about two years, and though not cured, still take a treatment occasionally when the eruption is troublesome. A fourth case did not appear to be influenced very much by injections of 75 to 150 c. c. of fresh sea water twice a week during several months.

A rare case of dermatitis gangrenosa with gangrenous patches on the abdomen, breast, neck and thigh in a thin, neurotic, poorly nourished girl of 20, was apparently cured after treatment irregularly for about a year. The gangrenous patches followed a carbolic acid burn of the finger five years before, and came and went at intervals of a few weeks for four years before she began this treatment. She was given doses of 75 to 150 c. c. twice a week.

In three cases of pityriasis rosea, the eruption began fading after the second or third injection of 50 to 75 c. c. of fresh sea water, and disappeared completely in three or four weeks.

In a case of tubercular wrist of 14 years' standing in a man 75 years of age, a complete cure was brought about in three months.

In some cases of extensive eczema I have noticed general improvement, but have not treated any case by means of sea water alone.

In a case of inveterate syphilis which was tabetic with gastric crises, and which was rebellious to both salvarsan and mercury, there was very marked and rapid improvement, not only in the tabetic symptoms but in the extensive papulo-squamous syphilide.

In several cases of advanced cancer, the pain was decidedly relieved by subcutaneous injections of sea water, and a temporary improvement in the general condition of the patient.

In conclusion I will say that theoretically sea water, the marine medium, being the original "vital medium" of animal cells, should, under proper precaution, be harmless if injected into the animal economy. In practice this is true. Except for the temporary reaction I have known of no serious results from its use.

In these days when we witness such marvelous, rapid and complete cures as are made through the blood by anti-toxic serums, such as that for diphtheria, anything which is not so absolutely certain in its results is apt to be regarded with indifference or skepticism. However, we have used other remedies in diphthria with success for many decades, so in many diseases which baffle us we must use anything which promises better results than we have had heretofore, even if it is not the specific we most strongly desire. Sea water alone or in combination with other treatment, will cure or materially improve many cases in the treatment of which we have hitherto been unsuccessful.

And in most cases where the results most desired are not secured, it greatly improves nutrition and the general condition, which is a long step forward.

It is a therapeutic agent which is deserving of more general recognition and investigation.

623 Stevens Building.

December 16th, 1912.

---

Glycyl Tryptophan Test For Gastric Carcinima.—Neubauer and Fischer, *Munch. Med. Woch.*, 1911, page 674, vol. 58, claim that normal gastric juice does not split this substance and that cancerous juice almost invariable does, basing their statement on a series in which 75% were verified clinically and 84% by necropsy. Austin, *Med. Rec.*, 1911, vol. 80, considers this test the most available clinically. On the other hand, Weinstein, *Jour. A. M. A.*, 1911, vol. 57 page 1420, holds that gastric contents practically always contain proetoses and peptones that are convertible by the cancer enzyme into tryptophan, among other cleavage products. I. Walker Hall and Scott Williamson, *Bristol Medico-chir. Jour.*, 1911, vol. 29, page 96, and Smithies, *Jour. A. M. A.*, 1912, vol. 59, page 539, think that the test may merely indicate the presence of blood serum which is, of course, especially frequent in cancer while, in ulcer, the presence of larger amounts of blood would obscure the test.

---

Hookworm Disease in Ancient Egypt.—Walter H. Page, *World's Work*, September, 1912, states that hookworm disease was described in Egypt in 1833, but that a very good clinical description of the disease has been found in a papyrus almost 3500 years old.

---

Chorion-Epithelioma of the Fallopian Tube.—Jeanneret, *Rev. Med. Suisse Rom.*, May 20, 1912, reports a case developing after extra-uterine pregnancy. He found only ten tubal cases in literature, the first by Marchand in 1895.

